# Supplementary material for: Pathophysiological Significance of Neutrophilic Transfer RNA-Derived Small RNAs in Asymptomatic Moyamoya Disease
Source: Cells. 2021 May 1;10(5):1086. doi: 10.3390/cells10051086 (PMC8147334; doi:10.3390/cells10051086)
Supplement: Supplementary file 1 [file cells-10-01086-s001.zip › cells-1149161 3rd supplementary materials/Data Supplements/Data Supplement V.zip!/Data supplement 6-mRNAs targets of tsRNAs/tsRNA-Val-AAC-017/Diagrams/tRF-Val-AAC-017_vs_ENST00000367455(UCHL5).pdf]

2D Structure	Local AU	Position
See alignment below	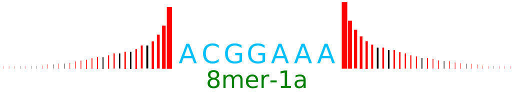	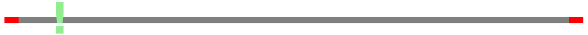

2D Structure

```
369                            400
                        8mer-1a
5'-ttgtcTTATTTCTTCTCATCATTAACGGAAAa-3' utr
        | ||  |: || | ||:| |||||||
3'-uuugcACUAUUGGUGA-U-GUGA-UGCCUUUg-5' tRF
                161514 13      765432
                3' Pairing      Seed
```

Local AU

Position
